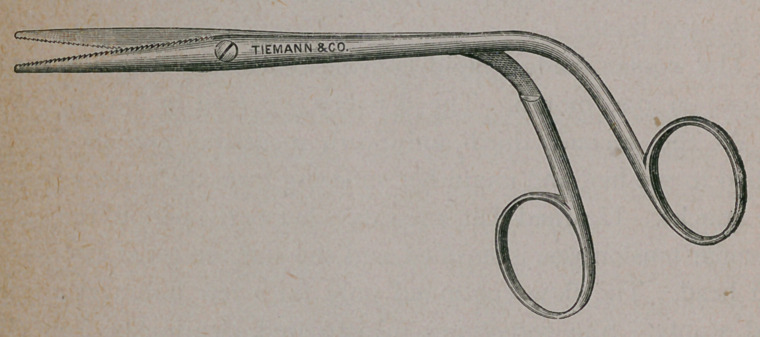# A Nasal Scissors

**Published:** 1888-03

**Authors:** Frank Hamilton Potter

**Affiliations:** Lecturer on Laryngology, Medical Department, Niagara University; 42 West Tupper Street


### *A NASAL SCISSORS.*

By FRANK HAMILTON POTTER, M. D.,

Lecturer on Laryngology, Medical Department, Niagara University.

The scissors can be used with great advantage in many operative procedures upon the nasal passages. The cut below illustrates a new form of the instrument, which, it is thought, possesses sufficient merit for publication.

Attention is called to the following points:

1. The hand of the operator is always below the line of vision, whether the instrument is open or shut.

2. The blades are one and five-eighths inches long, and will thus grasp large growths; or, with but a slight movement of the handles, they can be opened sufficiently to trim the edges of wounds. The latter point is well illustrated in the cut.

3. The cutting edges are serrated, so as to make an uneven wound, and thus favor the coagulation of blood.

4. It is strongly made, and thus allows, when necessary, the use of considerable force.

Other scissors have been devised embodying some of the points mentioned above, but it is believed that this instrument has so combined them as to increase the practical value of the scissors in nasal surgery.

It is made by Messrs. Tiemann & Co., of New York, from whom it can be obtained.

42 West Tupper Street.

## Figures and Tables

**Figure f1:**